# Molecular Mechanisms of Pituitary Cell Plasticity

**DOI:** 10.3389/fendo.2020.00656

**Published:** 2020-09-10

**Authors:** Gwen V. Childs, Angus M. MacNicol, Melanie C. MacNicol

**Affiliations:** Department of Neurobiology and Developmental Sciences, University of Arkansas for Medical Sciences, Little Rock, AR, United States

**Keywords:** pituitary, plasticity, multihormonal cells, multipotential, leptin, mRNA translation, Musashi, single cell

## Abstract

The mechanisms that mediate plasticity in pituitary function have long been a subject of vigorous investigation. Early studies overcame technical barriers and challenged conceptual barriers to identify multipotential and multihormonal cell populations that contribute to diverse pituitary stress responses. Decades of intensive study have challenged the standard model of dedicated, cell type-specific hormone production and have revealed the malleable cellular fates that mediate pituitary responses. Ongoing studies at all levels, from animal physiology to molecular analyses, are identifying the mechanisms underlying this cellular plasticity. This review describes the findings from these studies that utilized state-of-the-art tools and techniques to identify mechanisms of plasticity throughout the pituitary and focuses on the insights brought to our understanding of pituitary function.

## Introduction

**Figure 1:**
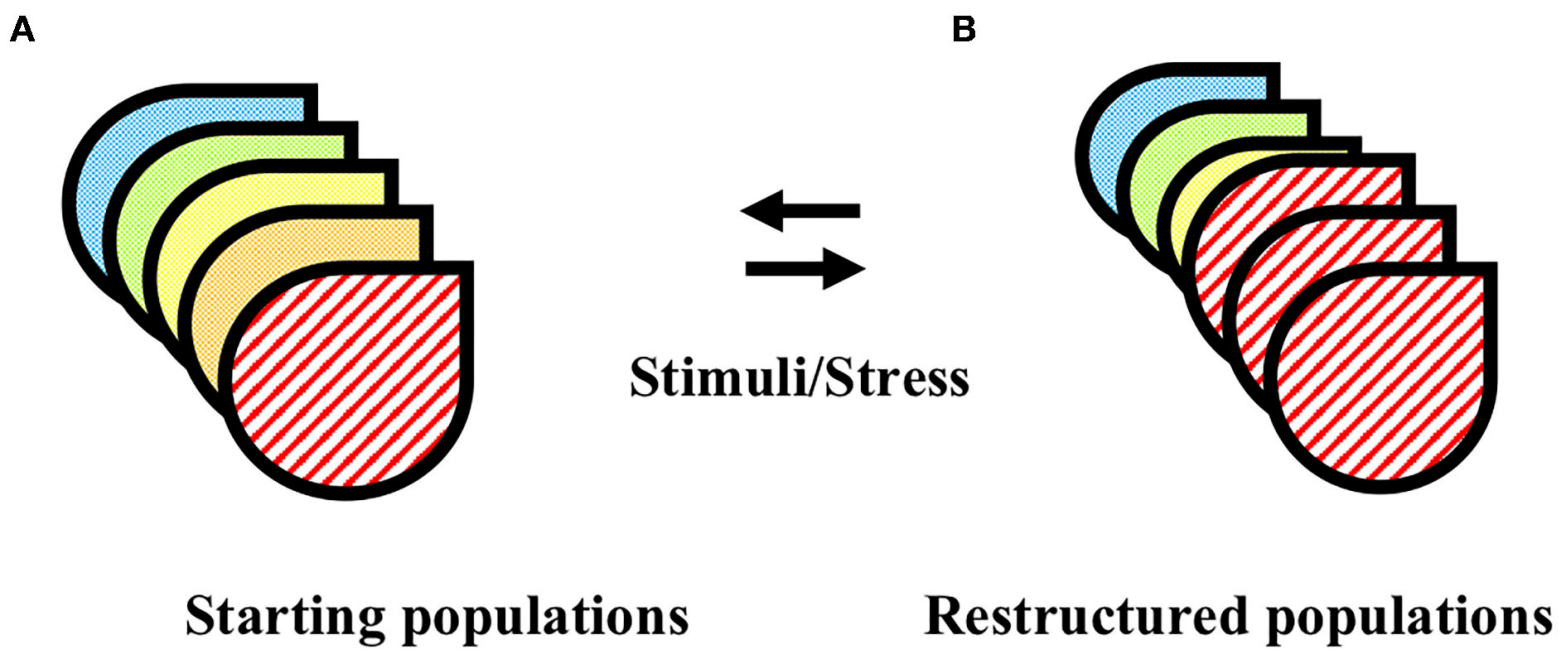
Pituitary cell populations are restructured in response to physiological stimuli and pathological stressors. **(A)** The pituitary is comprised of multiple distinct functional cell types (designated by different image fill patterns). **(B)** In response to physiological stimuli or pathological stress, the pituitary cell populations undergo restructuring. The double arrows imply the potential reversibility of this process. See text for details and relevant references.

The pituitary system orchestrates appropriate behavioral responses to fluctuating physiological and/or pathological signals, through controlled production and secretion of diverse signaling peptide hormones. The levels of hormone that must be secreted to meet effective serum levels for the intended response, is vast relative to the small size of the pituitary itself, and this defined size limits the number of cells that can be utilized in effecting any one of the many responses. These opposing challenges of limited cell numbers and diverse, large required outputs are resolved through plasticity in allocation of cell resources to each particular function (see Figure 1). The mechanisms controlling pituitary cell plasticity are a source of tremendous interest since these mechanisms first began to be revealed and evidence of malfunction in pituitary plasticity under diverse genetic and disease states has further motivated study to understand these mechanisms [for reviews see (1, 2)]. Over the last several decades, an ongoing procession of cutting edge, novel techniques have been developed and embraced in the effort to understand pituitary function and plasticity. In this mini review, we identify the multiple fortuitous links that have occurred between the need for information about a particular aspect of pituitary function and the identification, discovery and/or development of a new technique or tool that provided that information. We follow the pathway of discovery made with early cell microscopy technologies through the ongoing development and utilization of complex and specific animal models to the emerging utilization of molecular and computational techniques that together identify cell-specific information. We will primarily focus on discoveries made through those tools and techniques that we have utilized ourselves, with the expectation that this overview will give a representative example of the pathways that have led to our current understanding of pituitary plasticity.

## Breakthrough Discoveries; Visualization Leading to Cell Identification

**Figure 2:**
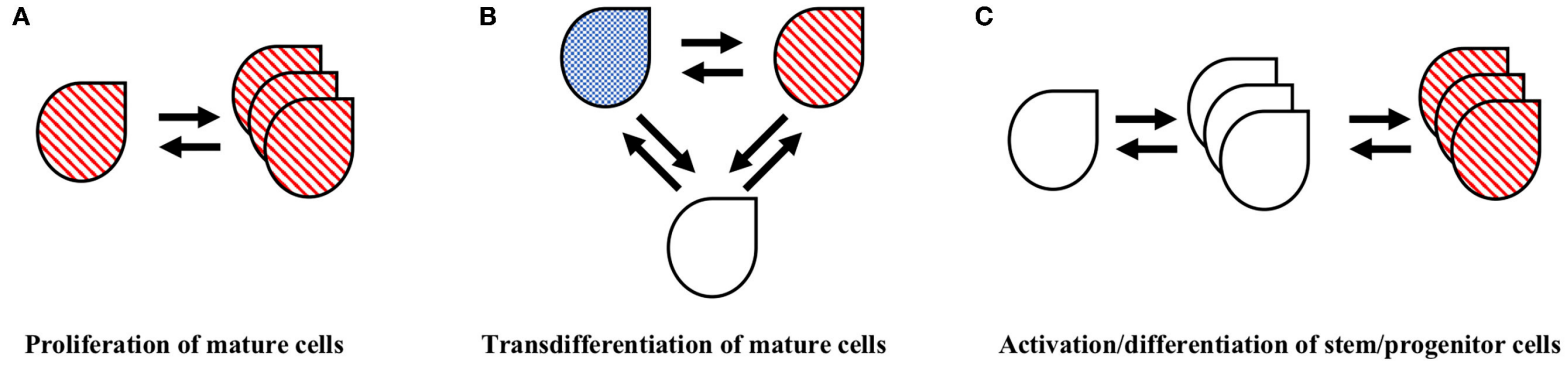
Cellular mechanisms that may contribute to pituitary population restructuring. There is evidence that pituitary populations undergo restructuring through multiple cellular mechanisms including **(A)** mitotic proliferation of existing hormone cells, **(B)** transdifferentiation of mature hormone cells and **(C)** activation and differentiation of stem/progenitor cells. The double arrows imply a potential reversibility of these cellular processes. See text for details and relevant references.

The development of multiplexing technologies, simultaneously identifying multiple proteins and/or both proteins and mRNAs in the same cell population enabled a conceptual breakthrough in identification of the mechanisms of pituitary plasticity. The identification of more than one hormone in a single cell or the identification of cell surface activating receptors and distinct intracellular hormones in the same cell, suggested the presence of multipotential cells, a novel concept in the face of an existing paradigm of pituitary organization that indicated each hormone is produced by a dedicated cell population (3). The experimental proof of *bona fide* multipotential cells, as opposed to the uptake of proteins to one cell type from another cell type, required development and utilization of complex staining protocols along with novel microscopy technologies (4). These early studies, including those utilizing biotin-streptavidin directed staining, *in situ* hybridization, and immunogold electron microscopy, enabled identification of specific populations within the mature adult pituitary that express the mRNA, receptors and/or hormones indicative of multiple distinct cell types. Studies using calcium signaling identified populations of pituitary cells that appeared to be fully differentiated toward a specific cell type, yet retained the capacity to respond to multiple releasing hormones of other distinct cell types (5). These findings introduced the concept of multihormonal/multipotential cells within the adult pituitary, with the capacity to contribute to functional plasticity through generation of whichever cell type was needed to serve hormone demand. The origin of these multipotential cells continues to hold fascination in the study of pituitary plasticity. The mechanisms by which they arise, either from an existing subpopulation of immature progenitor cells or through a transdifferentiation of existing hormone-producing, mature cells remains an open question (6–8) (see Figure 2).

As the identification of multipotential/multihormonal cell types was hampered by technical barriers in distinguishing these cells from their monohormonal counterparts, so conceptual barriers further occluded their identification. Models of pituitary organization based upon embryological studies showed the ventral to dorsal gradients of tissue differentiation factors including Bmp2 and Gata2, that stimulate generation of gonadotropes in ventral regions and restrict expression of the opposing differentiation factor Pou1f1 (also named Pit-1), which is required for the generation of somatotropes, thyrotropes and lactotropes (9, 10). The decreased levels of Bmp2 in dorsal regions is thus required to allow the differentiation of somatotropes as Pou1f1 binds to Gata2 and prevents it from activating factors needed for gonadotrope development. These findings together support a model in which monohormonal somatotropes and gonadotropes develop in separate regions of the embryonic pituitary with the balance between expression of Pou1f1 and Gata2 restricting specification of each cell type (11–13). This developmental model appeared to preclude the presence of cells that produce multiple hormones, such as those that would require both Pou1f1 and Gata2, e.g., somatotogonadotropes (14).

Continued efforts in the field, however, revealed a model in which pituitary plasticity utilizes all members of a cell population, including multihormonal/multipotential subtypes, in response to challenges by stimuli that require multiple hormone responses. This coordinated multi cell-type response was demonstrated through affinity cytochemical studies showing the estradiol-mediated increase in gonadotropin releasing hormone receptor (GnRHR) production by somatotropes, as well as by gonadotropes, with the somatotropes defined by their expression of growth hormone (GH) protein and mRNA (15). Gonadotropin releasing hormone (GnRH) was thus found to stimulate an increase in production of the “gonadotrope-specific” hormones, luteinizing hormone (LH) and follicle-stimulating hormone (FSH) by both gonadotropes and a subset of somatotropes/somatogonadotropes to support the high levels of LH and FSH that are needed to effect estrous cycle surges. A role for multihormonal cells in mediating pituitary plasticity was further proposed to facilitate the high levels of adrenocorticotropic hormone (ACTH), beta-endorphin, and thyroid stimulating hormone (TSH) that are required for the response to extreme or prolonged cold (16). The coordination of these multihormonal responses appear to require contributions from cells capable of producing multiple hormones in response to distinct neuropeptide secretagogues as has been demonstrated through cold stress-induced, arginine vasopressin (AVP) stimulation of “thyrocorticotropes” to produce both ACTH and TSH (17, 18). As these cells also respond to thyrotropin-releasing hormone (TRH) and corticotropin-releasing hormone (CRH), both of which enhance AVP receptor expression, these multipotential target cells would serve to amplify the pituitary response to stress.

The identification of mechanisms underlying pituitary functional plasticity has benefitted from the development and use of a number of techniques to isolate distinct pituitary cell subpopulations. These are reviewed in a recent publication (19). One early method developed in our laboratory included counterflow elutriation that took advantage of the different size and morphologies of distinct pituitary cell types to obtain subpopulations that were 90% pure (20). However, higher levels of purity are obtained with approaches that involve the expression of cell type-specific fluorescent proteins such as the enhanced green fluorescent protein (eGFP), linked to the gene promoter of a cell-type specific protein, such as somatotrope GH, enabled the use of fluorescent activated cell sorting technologies (FACS) that provide a pure population of live cells for downstream analyses (21). Development of pituitary cell-lineage tracing mouse models have identified a population of *Sox2*-expressing stem cells in the adult pituitary that can differentiate to produce all hormone-expressing cell types (22–26). Although activation of this cell population has been observed under conditions of acute experimental stress, e.g., hormone cell type-specific cell oblation and organ loss, the extent to which this mechanism contributes to pituitary homeostasis and functional plasticity is unclear (27). Adult stem cells have also been implicated in mediating pituitary neo-plastic growth, and this pathological aspect of pituitary plasticity has been extensively covered in recent excellent reviews (28, 29).

Continuing developments in microscopy, including the use of live cell imaging and electrophysiological tools, along with the development of analysis software has further enabled the identification of pituitary cell morphology plasticity and the observation of pituitary cell process motility and remodeling (30). Together, these cell properties have been shown to contribute to the formation of complex, three-dimensional heterotypic and homotypic pituitary cell networks that functionally contribute to plasticity of response (31, 32). Identification of the distinct subpopulations within a specific hormone cell type has been further facilitated through use of teleost transgenic models that demonstrate the role of heterotypic network communication in mediating gonadotrope function and plasticity (33). The ongoing development of pituitary tissue and cell culture techniques and the *in vitro* growth of pituitary stem/progenitor cells as “organoids” is directed toward development of an experimental model that is being utilized to address mechanisms of pituitary function that occur on an intermediate timescale, between the short term of cell culture and the lifetime of animal models (34–36). Organoid model developments include the use of human induced pluripotent stem cells (iPSCs) and the co-differentiation of hypothalamic and pituitary tissues in patient-specific organoids (37, 38). These human cell-based models complement the use of transgenic models in revealing the mechanisms underlying pituitary cell plasticity.

## Continuing Discoveries; Mechanisms Mediating Cell Plasticity

The utilization of transgenic animal models has greatly facilitated the study of pituitary function and plasticity. Many diverse, genomic models have been employed in identification of the roles of specific mediators of pituitary physiology and disease through the identification of effects upon whole animal physiology [for review see (2)]. Through the use of genetic models of pituitary cell type-specific leptin receptor knock-out, we have identified the mechanisms by which energy stores, as indicated through serum leptin signaling, influence pituitary function to optimize growth and reproduction. Findings from these studies have revealed the direct influence that leptin signaling has upon pituitary cell plasticity and the maturation required for hormone protein synthesis and secretion from pituitary somatotropes and pituitary gonadotropes (19, 39). Findings from these studies include the observation of decreased levels of growth hormone gene transcription (*Gh* mRNA) in pituitary somatotropes under conditions of loss of leptin signaling to the pituitary, thus indicating a link between leptin signaling to the pituitary and specific activator (s) of the *Gh* gene regulatory machinery (19).

**Figure 3:**
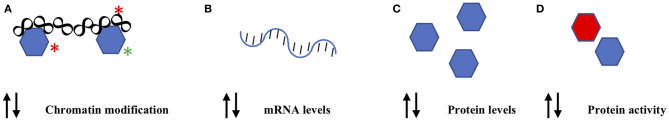
Molecular mechanisms that may contribute to pituitary population restructuring. Pituitary cell populations may utilize multiple molecular mechanisms for restructuring including **(A)** chromatin remodeling **(B)** gene transcriptional control **(C)** control of mRNA translation to protein and **(D)** control of protein function. The double arrows indicate the potential for both activating (positive) and inhibitory (negative) effects upon these molecular mechanisms. See text for details and relevant references.

Gene expression detection techniques have further resulted in the discovery of the absence of altered gene transcription under conditions in which it is expected, e.g., under conditions in which an increase in levels of GnRHR is observed in pituitary gonadotropes in response to leptin stimulation, that is not coincident with an increase in *Gnrhr* mRNA (39). This observed lack of concordance between changes in protein levels and changes in cognate mRNA levels suggests that a post-transcriptional mechanism mediates this regulatory process (40). We have recently found that the translation of the *Gnrhr* mRNA is repressed through the action of the RNA binding protein Musashi1 through direct association with the *Gnrhr* mRNA 3′untranslated region (41). Leptin stimulation is proposed to inhibit Musashi1 function, allowing de-repression and translation of the *Gnrhr* mRNA (39, 41). A recent study has demonstrated an opposing role for the RNA binding protein ELAVL1 through the post-transcriptional enhancement of *Gnrhr* mRNA stability (42). Since Musashi1 and ELAVL1 are found in common mRNA ribonucleoprotein complexes, Musashi1 and ELAVL1 may be coordinately regulated to differentially govern *Gnrhr* mRNA translation and thus gonadotrope remodeling throughout the estrus cycle (43). Several recent transcriptomics analyses of the pituitary at the cell type-specific and at the single cell level, have revealed an extraordinary level of variation in cell identity (44–47). The potential for plasticity at the cellular level, as defined by expression of genes associated with multiple hormone-producing cell types has been identified in a significant percentage of adult pituitary cells (47). These technologies greatly contribute to the comprehensive development of a model of pituitary cell functional plasticity. Findings from these analyses have definitively identified a multihormonal-expressing population within the adult pituitary that undergoes a high level of plasticity in hormone gene expression in response to the physiological stresses (47). The relevance of these data to mechanisms of pituitary plasticity will continue to be revealed as new ‘omics data are obtained and bioanalysis tools are developed (48, 49) (see Figure 3).

## Conclusions and Future Directions; Big Data to Molecular Manipulation

**Figure 4:**
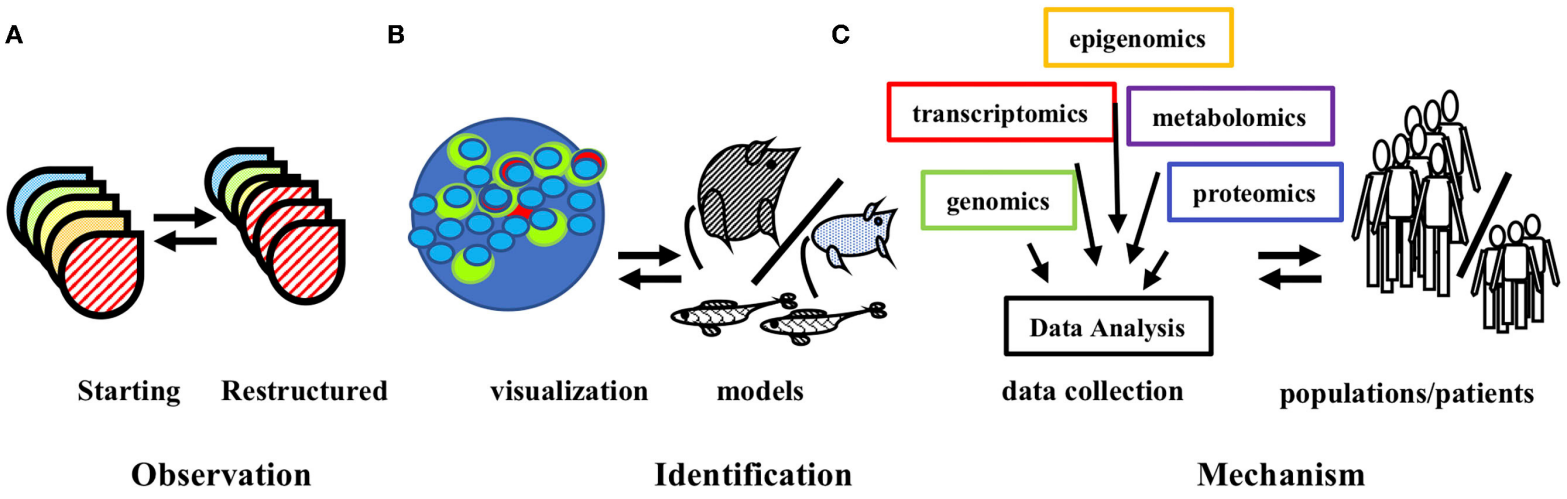
Technological innovations contribute to understanding pituitary plasticity. **(A)** Early observations identified remodeling of pituitary hormone cell populations in response to stimuli and stress. **(B)** Microscopic observations and genetic models identified the cell populations that mediate restructuring. **(C)** The complimentary use of diverse data set analyses provides insight into the molecular mechanisms of pituitary population restructuring and suggest targets for therapeutic options for patients. The double arrows imply the information feedback of these complimentary technologies. See text for details and relevant references.

The understanding of pituitary function is relevant to diverse biomedical fields. Pituitary plasticity is fundamental to reproductive and endocrine function and impinges upon the control of metabolic disease, cancer and cell replacement therapies. From early imaging and cell morphology assays to current ‘omics analyses, the array of methodologies directed toward an understanding of pituitary function stands as an exemplar of biomedical research capabilities (see Figure 4). The study of pituitary plasticity continues to utilize state of the art and emerging tools that provide data relevant to endocrine function and disorders over a wide biological scale from whole population patient data sets to molecular mechanisms revealed by novel animal models and single cell analyses. Ongoing and future studies are expected to combine targets revealed through these analyses with experimental interventions (35, 50). The recognition of the limitations of existing methods has driven the development and use of novel technologies in pursuit of an understanding pituitary function. Similar to the creative courage that is required to propose new hypotheses, so, experimental courage is often required to test them; the employment of these together, will ensure that our ongoing investigations of pituitary function and plasticity will continue in new and exciting directions.

## Author Contributions

GC and MM planned and wrote the paper. MM designed the figures. GC, AM, and MM edited the paper. All authors contributed to the article and approved the submitted version.

## Conflict of Interest

The authors declare that the research was conducted in the absence of any commercial or financial relationships that could be construed as a potential conflict of interest.
